# HMA6 and HMA8 are two chloroplast Cu^+^-ATPases with different enzymatic properties

**DOI:** 10.1042/BSR20150065

**Published:** 2015-06-11

**Authors:** Emeline Sautron, Hubert Mayerhofer, Cécile Giustini, Danièle Pro, Serge Crouzy, Stéphanie Ravaud, Eva Pebay-Peyroula, Norbert Rolland, Patrice Catty, Daphné Seigneurin-Berny

**Affiliations:** * CNRS, Laboratoire de Physiologie Cellulaire et Végétale, UMR 5168, 17 rue des Martyrs, F-38054 Grenoble, France; † Univ. Grenoble Alpes, F-38054 Grenoble, France; ‡ CEA, DSV, iRTSV, F-38054 Grenoble, France; § INRA, LPCV, USC1359, 17 rue des Martyrs, F-38054 Grenoble, France; ║ CEA, DSV, Institut de Biologie Structurale, F-38044 Grenoble, France; ¶ CNRS, Institut de Biologie Structurale, UMR5075, 71, avenue des Martyrs, F-38044 Grenoble, France; * *CNRS, Laboratoire de Chimie et Biologie des Métaux, UMR 5249, 17 rue des Martyrs, F-38054 Grenoble, France

**Keywords:** Arabidopsis, chloroplast, copper, P_IB_-ATPase, plant biochemistry, thylakoids, transporter

## Abstract

Copper (Cu) plays a key role in the photosynthetic process as cofactor of the plastocyanin (PC), an essential component of the chloroplast photosynthetic electron transfer chain. Encoded by the nuclear genome, PC is translocated in its apo-form into the chloroplast and the lumen of thylakoids where it is processed to its mature form and acquires Cu. In *Arabidopsis*, Cu delivery into the thylakoids involves two transporters of the P_IB-1_ ATPases family, heavy metal associated protein 6 (HMA6) located at the chloroplast envelope and HMA8 at the thylakoid membrane. To gain further insight into the way Cu is delivered to PC, we analysed the enzymatic properties of HMA8 and compared them with HMA6 ones using *in vitro* phosphorylation assays and phenotypic tests in yeast. These experiments reveal that HMA6 and HMA8 display different enzymatic properties: HMA8 has a higher apparent affinity for Cu^+^ but a slower dephosphorylation kinetics than HMA6. Modelling experiments suggest that these differences could be explained by the electrostatic properties of the Cu^+^ releasing cavities of the two transporters and/or by the different nature of their cognate Cu^+^ acceptors (metallochaperone/PC).

## Abstract (precis)

In *Arabidopsis* chloroplasts, HMA8, the thylakoid Cu^+^-ATPase and HMA6, the envelope Cu^+^-ATPase, have different enzymatic properties. These differences might be related to the electrostatic properties of the Cu^+^ release cavities of the transporters or the nature of their Cu acceptor.

## INTRODUCTION

Photosynthesis is the fundamental process by which molecular oxygen is generated in the earth's biosphere. This reaction takes place in the chloroplasts of photosynthetic organisms and involves soluble and membrane proteins present in the thylakoids. Copper (Cu) plays a key role in the photosynthetic process being the redox metal of plastocyanin (PC), a small soluble protein required for electrons transfer from the cytochrome *b_6_/f* to the photosystem I [1]. The two *Arabidopsis* PC isoforms, PETE1 and PETE2, are involved in the photosynthetic electron transport [2]. PETE2, whose expression is regulated by intracellular Cu concentration is the more abundant PC isoform and was suggested to also behave as a Cu sink in the presence of Cu excess. PETE1 is commonly described as the PC isoform that drives electron transport under Cu-deficiency. The latter isoform is less abundant and is not regulated by Cu concentration [3]. Since PC is essential for photosynthesis, Cu delivery to the thylakoids is a priority for plants grown in autotrophic conditions [4]. In the *Arabidopsis* chloroplast, Cu is not only essential for photosynthesis but also for the activity of the Cu/Zn superoxide dismutase (Cu/Zn-SOD), a soluble enzyme that scavenges reactive oxygen species produced by photosynthesis under stress conditions [5].

Its chemical properties make Cu a key element in redox reactions but also a toxic compound when present in excess in the cell. Therefore, assimilation and distribution of Cu must be tightly regulated to fit cellular requirements. To that aim, a complex network of uptake, chelation, trafficking and storage processes ensures Cu homoeostasis in all cells. Cu transporters, among them P_IB_-ATPases and their cognate metallochaperones, are essential components of this network. P_IB_-ATPases [6] belong to the large family of P-ATPases, transmembrane (TM) proteins responsible for the transport of ions and phospholipids across plasma and organelle membranes using the energy provided by ATP hydrolysis. The catalytic cycle of P_IB_-ATPases can be schematically described as a four-step process (Supplementary Figure S1). In the first step, a cytosolic metal ion binds to the high-affinity transport site in the TM domain of the transporter. Ion binding allows the phosphorylation from ATP of a conserved aspartate residue in the large cytosolic domain of the P_IB_-ATPase (Supplementary Figure S1, step 2). The so-formed E~P.Me intermediate undergoes conformation changes leading to metal release at the extracytosolic side and the formation of E-P, a metal-free phosphorylated intermediate (Supplementary Figure S1, step 3). The aspartyl-phosphate bound is then hydrolysed to bring the P_IB_-ATPase back to its initial state (Supplementary Figure S1, step 4).

Like all P-ATPases, P_IB_-ATPases are made of a TM and an extra-membranous part. The former (M-domain) contains the transport site and determines ion selectivity. According to the presence of conserved residues in the TM segments TM6, TM7 and TM8, P_IB_-ATPases have been classified into five subgroups, IB-1–5 of distinct metal specificities [7]. P_IB-1_-ATPases are characterized by the conserved sequences C-P-C in TM6, Y-N, in TM7 and M-X-X-S-S in TM8 and comprises all Cu^+^-ATPases such as the bacterial CopAs (copper-resistance operon protein A) from *Escherichia coli* [8], *Enterococcus hirae* [9], *Archeoglobus fulgidus* [10] and *Legionella pneumophila* [11], but also Ccc2p (Ca^2+^-sensitive cross-Complementer protein) from *Saccharomyces cerevisiae* [12] and the human Cu^+^-ATPases ATP7A and ATP7B [13–15]. P_IB-4_-ATPases are characterized by the conserved sequences S-P-C in TM6 and H-E-X-G in TM8 and were described as Co^2+^-, Zn^2+^-, Cd^2+^- or Ni^2+^-ATPases [16,17]. Note that a mutation in one or the other of the two cysteines of the C-P-C motif prevents metal binding leading to non-functional proteins [12,18]. The extra-membranous part of the transporter is made of three domains called N for ‘nucleotide-binding domain’, P for ‘phosphorylation domain’ (this domain contains the conserved aspartic acid residue transiently phosphorylated within the catalytic cycle) and A for ‘actuator domain’. Most of the P_IB_-ATPases have additional N- and/or C-terminal metal-binding domains (MBD).

In *Arabidopsis*, Cu delivery to chloroplasts and thylakoids requires three P_IB_-ATPases: heavy metal associated protein 1 (HMA1), HMA6 and HMA8 [19]. From sequence analysis, HMA6 and HMA8 belong to the subgroup IB-1; HMA1 however displays the characteristics of P_IB-4_-ATPases. The main phenotypes of the *Arabidopsis hma6*, *hma8* and *hma1* mutants, listed in the Supplementary Table S1, suggest that (i) HMA6, located in the chloroplast envelope, is the main Cu delivery pathway to the stroma [20]; (ii) HMA8, located in the thylakoid, delivers Cu to PC [21]; (iii) HMA1, essential under light stress conditions, is also involved in Cu import into the chloroplast [22,23] but might also transport other metal ions [24,25]; (iv) HMA1 and HMA6 behave as distinct pathways for Cu delivery into the chloroplast [23]; (v) a third low-affinity Cu import system exists in the chloroplast envelope [23]. In a recent work, Blaby-Haas et al. [26] have shown that two different metallochaperones namely PCH1 (plastid chaperone 1) and CCS (copper chaperone for SOD), interacted *in vitro* with the N-terminal truncated and purified HMA6 and HMA8 respectively. In that study, these forms of HMA6 and HMA8 were shown to display identical ATPase activities and similar affinities for Cu^+^.

In the present paper, we have compared the enzymatic properties of the full-length HMA6 and HMA8 proteins after heterologous expression in *Lactococcus lactis*. Corroborated by observations in yeast, our biochemical results obtained from phosphorylation assays, highlight enzymatic differences between the two chloroplast Cu^+^-ATPases, HMA6 and HMA8. Using modelling and docking experiments, we proposed that these differences might be related to the electrostatic properties of the Cu^+^ release cavities of the transporters and/or the nature of their Cu acceptor.

## EXPERIMENTAL

### HMA8 and HMA6 expression in *Lactococcus lactis*

HMA8 sequence (At5g21930) used in the present study codes for the mature form of the protein, i.e. lacking the 65 first amino acid residues corresponding to the predicted chloroplast transit sequence (ChloroP, [27]). The cDNA was PCR-amplified from an *Arabidopsis* cDNA library using the primers *Sph*I-Nter (5′-CAGCGCATGCTCATGATCGAATCTGTG-3′) and *Xba*I-Cter (5′-CTGAAACAAGTAAAAATAGCTTGTGGTCACACCCA-CAATTCGAGAAG TGATCTAGATCG-3′). The PCR product was cloned into the pBluescript KS^−^ plasmid (Stratagene) and further inserted into pNZ8148, a nisin-inducible vector for expression in Lactococcus. Vectors were purified by MiniPrep (Macherey-Nagel). Sequencings were performed at Eurofins MWG Operon. The expression of HMA8 and HMA6 (At4g33520) from the pNZ8148 vector as well as the preparation of bacterial membranes were performed as previously described [28,29]. Site-directed mutagenesis (Quickchange®, Stratagene) was performed on the pKS-HMA8 plasmid to produce the D548A mutant (hereafter named HMA8-AKT, amino acids Alanine, Lysine and Thréonine) and the C504A/C506A mutant (hereafter named HMA8-APA, amino acids Alanine, Proline, Alanine). All the proteins expressed in Lactococcus contain a *Strep*-Tag II (IBA) fused at their *C*-terminal end.

### SDS/PAGE and detection

Protein content was estimated using the Bio-Rad protein assay (Bio-Rad). SDS/PAGE analyses were performed as described by Chua [30]. Detection of the *Strep*-Tag II was performed using the *Strep*-Tactin HRP (horseradish peroxidase) conjugate (IBA) at a 1:10000 dilution, followed by ECL detection.

### Phosphorylation assays

Phosphorylation assays were performed as previously described [29] in 100 μl of 20 mM HEPES (pH 6.0 or pH 7.0), 100 mM KCl, 10 mM MgCl_2_, 300 mM sucrose, 500 μM of the reducing agent Na_2_SO_3_ and either 50 μg of membranes containing HMA6 (or HMA6-AKT) or 150 μg of membranes containing HMA8 (or HMA8-AKT and HMA8-APA). To minimize contamination with metals, HEPES and sucrose were treated with Chelex (5 g/100 ml, 1 h under gentle stirring). Phosphorylations were performed at room temperature or at 4°C in the presence of various concentrations of metals or chelators [1 mM BCA (bicinchoninic acid), 0.1 mM BCS (bathocuproine disulfonate), 1 mM EGTA] either alone or in combination. The reaction was started by addition of 1 μM [γ-^32^P]-ATP (50–500 μCi nmol^−1^) and the sample treated as described in Catty et al. [29]. The phosphorylation signal was revealed using a phosphorimager (Cyclone, PerkinElmer Life Science) and analysed using the Optiquant Software (PerkinElmer Life Sciences). The amount of loaded proteins was checked after Coomassie Blue staining.

### Expression and phenotypic tests in *Saccharomyces cerevisiae*

HMA8, HMA8-AKT and HMA6 were expressed as described [29] from a centromeric vector under the control of the constitutive and strong PMA1 (promoter of the yeast plasma membrane ATPase1) promoter. For confocal imaging, HMA8, HMA8-AKT and HMA6 sequences have been fused to the GFP encoding sequence. For maintenance, the *S. cerevisiae* strain BY4741 [31] was grown at 30°C in rich YD (yeast dextrose) medium [1% (w/v) yeast extract KAT (trade mark), 2% (w/v) glucose]. Cells were transformed as described in Kuo and Campbell [32]. Plasmid selection was performed on solid synthetic minimal medium containing 2% (w/v) glucose, 0.17% (w/v) yeast nitrogen base, 0.5% (w/v) ammonium sulfate 0.7 g/l dropout powder without leucine and 2% (w/v) Agar-Y (Bio 101® Systems). Drop-test experiments were performed using CuSO_4_ at various concentrations as described in Figure 1 legend.

**Figure 1:**
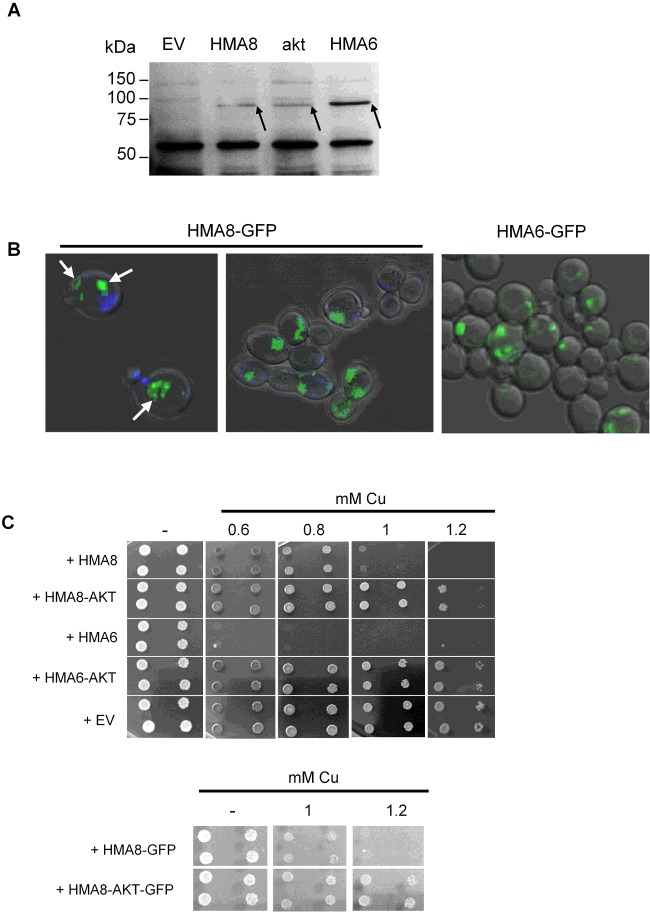
Functional expression of HMA8 in yeast (**A**) Expression of HMA6, HMA6-AKT, HMA8 and HMA8-AKT in yeast membranes was revealed by western blot using a *Strep*-tactin HRP conjugate. (**B**) Intracellular localization of HMA8-GFP and HMA6-GFP in yeast was determined by confocal fluorescence microscopy. The GFP signal (green fluorescence) is indicated by the arrows. Nuclei (blue) are stained with DAPI. (**C**) Yeast strains expressing HMA6, HMA8, HMA8-GFP or non-functional mutants (HMA6-AKT, HMA8-AKT, HMA8-AKT-GFP) were grown on selective media supplemented with Cu at the indicated concentration. For each expression condition, two independent transformants were spotted at dilutions 1 and 1/10, as 2 μl of drops. Dilution 1 corresponds to an optical density of 1.5 at 600 nm. **Abbreviation:** EV, empty vector.

### Yeast membrane preparation

A 24-h preculture of cells expressing HMA8 (or HMA8-AKT/HMA8-GFP/HMA8-AKT-GFP/HMA6) was used to inoculate a 100 ml of selective medium. After 24 h at 30°C, cells were spun down (5 min/5000 ***g***/4°C), washed with ice-cold water, resuspended in ice-cold buffer A [50 mM HEPES (pH 6.0), 100 mM KCl, 1 mM MgCl_2_, 10% (w/v) glycerol, protease inhibitors cocktail) and lysed by passage through a One Shot (Constant Cell Disruption Systems) at 35000 p.s.i. (1 lbf/in2≈6.9 kPa; 2.4 kbars). The lysate was centrifuged (1000 ***g***/10 min/4°C) to remove unbroken cells and large debris and the resulting supernatant was centrifuged at 100000 ***g*** for 50 min, at 4°C. The pellet, corresponding to the membrane fraction, was resuspended in 200 μl of ice-cold buffer A and stored at −80°C.

### Microscopy

Yeast cells were analysed by confocal laser scanning microscopy (Leica TCS-SP2 operating system). Observations were done with a 40× oil immersion objective. For nuclei detection, yeast cells in the mid-log phase were incubated with 1 μg/ml of DAPI for 30 min at 30°C, washed twice in PBS and suspended in water. GFP and DAPI were excited at 490 and 405 nm respectively. The fluorescence was collected from 500 to 535 nm for GFP and from 432 to 474 nm for DAPI. Transmission images where recorded using differential interference contrast optics.

### Measurements of metal ions content

Samples exactly reproducing the phosphorylation conditions were mineralized [in 65% HNO_3_/30% HCl, 2/1 (v/v) followed by evaporation at 180°C] and later dissolved in 10% (v/v) HNO_3_. Elemental analysis was performed on an inductively coupled plasma mass spectrometer (ICP-MS; Hewlett-Packard 4500 Series, Agilent Technologies) equipped with a Babington nebulizer and a Peltier-cooled double-pass Scott spray chamber. The device was calibrated at *m/z* 63 and 65 for Cu using standard solutions in 1% HNO_3_. ICP-MS analyses were performed after appropriate sample dilution with 1% HNO_3_. Quantification results are the mean values obtained for isotopes 63 and 65 obtained in four independent measurements.

### Determination of electrostatic potential maps and docking simulations

Sequences were taken from Uniprot entries ‘HMA6_ARATH’ and ‘HMA8_ARATH’. The MBD extends from Ile^149^ to Pro^223^ for HMA6 and from Pro^77^ to Arg^147^ for HMA8 whereas the TM part was modelled from Thr^237^ to Phe^920^ for HMA6 and from Met^164^ to Ser^876^ for HMA8. PC was modelled from Ile^1^ to Asn^99^. Initial models of HMA6 and HMA8 were built by homology modelling with the program Modeller [33] from the structure of LpCopA, the Cu-ATPase from *L. pneumophila* (PDB code 4BBJ) [11]. The blast sequence similarities between HMA6, HMA8 and LpCopA are 55% and 53% respectively, which allows a good confidence in the method. Fifty models of each protein were built with MODELLER and the model with lowest DOPE score retained. This model was itself refined with the MODELLER loop modelling protocol to yield the final model. PC from *Arabidopsis*, PETE2 isoform (Uniprot name PLAS2_ARATH) was modelled from the 1.08 Å (1 Å=0.1 nm) crystal structure of oxidized poplar PC at pH 4.0 (PDB code 4DP7) [34]. The 88% similarity between the two proteins allows a direct construction of an initial structure of PETE2 from the PDB structure. The co-ordinates of PETE2 were copied from those of the aligned amino acids in 4DP7 and missing side chain atom co-ordinates were built from internal co-ordinates with the molecular dynamics program CHARMM [35]. The electrostatic potential maps of each protein were computed with Adaptive Poisson-Boltzmann Solver software (APBS) [36] within visual molecular dynamics (VMD) [37]. The program HADDOCK [38] was used to dock PETE2 on HMA8. The Cu-binding site in PETE2 is constituted of amino acids His^37^, Cys^84^ and His^87^. Glu^189^ in LpCopA has been presented as a putative exit site for Cu whereas Met^717^ would be an intramembranous Cu-binding residue crucial for function [11]. The HMA8 corresponding amino acids in the sequence alignment are Asp^282^ and Met^857^. His^37^, Cys^84^ and His^87^ in PETE2 and Asp^282^, Met^857^ in HMA8 were consequently defined as active residues for HADDOCK meaning that the program will favour the presence of these residues at the docking interface.

## RESULTS

### Expression of HMA8 increases yeast sensitivity to Cu^+^

As previously shown for HMA6 [29], HMA1 [22,24,25] and the bacterial Cd^2+^-ATPase CadA (cadmium ATPase) [39], yeast is a useful tool for the functional characterization of P_IB_-ATPases. As shown in Figure 1(A), HMA8 and HMA8-AKT are produced in yeast but at a lower level than HMA6. Confocal imaging showed that HMA6 and HMA8, detected using GFP fusions, concentrated inside the cell in rather diffuse structures often close to the nucleus but never at the plasma membrane (Figure 1B). Such structures have already been reported for other P-ATPases expressed in yeast and were shown by EM to derive from endoplasmic reticulum (ER) [40,41]. For HMA8, phenotypic tests were carried out on the basis of earlier experiments showing that expression of HMA6 induced yeast growth arrest from 0.5 mM CuSO_4_ [29]. As shown in Figure 1(C), HMA8 (and HMA8-GFP) also increases yeast sensitivity to Cu but the growth arrest is observed at ~1.2 mM CuSO_4_, a concentration that has no effect on strains containing the empty vector or expressing non-functional transporters (HMA8-AKT, HMA8-AKT-GFP or HMA6-AKT). Intracellular localization data combined to the fact that Cu^+^ is the major form of Cu in yeast [42], strongly suggest that the observed phenotype results from ER overload with Cu^+^ due to HMA8 activity. The difference in sensitivity to Cu between HMA6 and HMA8 expressing strains might be related to the relative amount of the transporters in yeast or to their intrinsic enzymatic properties.

### Production of HMA8 in Lactococcus

The bacterium *L. lactis* was found particularly efficient for the production of three P_IB_-ATPases from *Arabidopsis thaliana*, HMA1, HMA3 and HMA6 [28]. The same expression conditions were used in the present study to express the mature form of HMA8, the *Arabidopsis* P_IB_-ATPase located at the thylakoid membrane. As shown in Figure 2 from western blot analysis using the *Strep*-tactin conjugate, HMA8 is produced in *L. lactis* but 5–10 times less than HMA6 (lane 4 compared with lanes 2 and 3), a situation very similar to what previously observed in yeast. The same low expression level was observed for two non-functional mutants of HMA8. One is HMA8-AKT, which contains an alanine residue instead of the aspartic acid residue phosphorylated within the transport process. The other is HMA8-APA, where the two cysteines constituting the membrane transport site in TM6 (C-P-C motif) have been replaced by alanine residues (Figure 2, lanes 5 and 6). This means that the low expression level of HMA8 in Lactococcus does not result from a toxic effect of the functional transporter that would lead to the selection of low expressing transformants, but rather from an intrinsic characteristic of the HMA8 gene. Our efforts to increase HMA8 expression level by changing the type of nisin (homemade compared with commercial), the nisin concentration, the induction time as well as the cell density at induction remained unsuccessful (result not shown).

**Figure 2:**
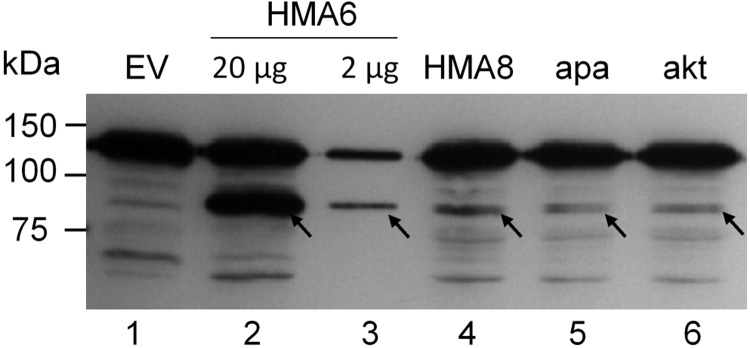
Production of chloroplast P_IB_-ATPases in Lactococcus membranes Total membrane proteins (20 μg in lanes 1, 2 and 4–6 or 2 μg in lane 3) were separated by SDS/PAGE. *Strep*-tag II present at the *C*-terminus of HMA6 and HMA8 was detected using a *Strep*-tactin HRP conjugate. The arrows indicate HMA6, HMA8, HMA8-AKT (akt) and HMA8-APA (apa). EV: membrane extract derived from bacteria containing the empty pNZ8148 vector.

### HMA8 is transiently phosphorylated from ATP

Prior to comparing the enzymatic properties of HMA6 and HMA8, we first checked that HMA8 was active in Lactococcus membranes. For that, we performed phosphorylation from [γ-^32^P]-ATP in the same conditions as those previously used for HMA6 [29]. In the presence of 1 μM added Cu^+^ (obtained by the use of 1 μM CuSO_4_ in the presence of 500 μM of the reducing agent Na_2_SO_3_), a phosphorylated band of the expected size (88.5 kDa) was observed in membranes containing HMA8 (Figure 3A) but not in membranes containing HMA8-AKT or HMA8-APA. In these latter, three endogenous radioactive signals of lower intensities than HMA8 were detected, one very faint at approximately the same size as HMA8 and two other more intense above and below it. In the same experimental conditions, the phosphorylated form of HMA6 (90.5 kDa) appears at a higher molecular mass than HMA8 and with a higher intensity, as expected from the relative amount of the two proteins in Lactococcus membranes (Figure 3A).

**Figure 3:**
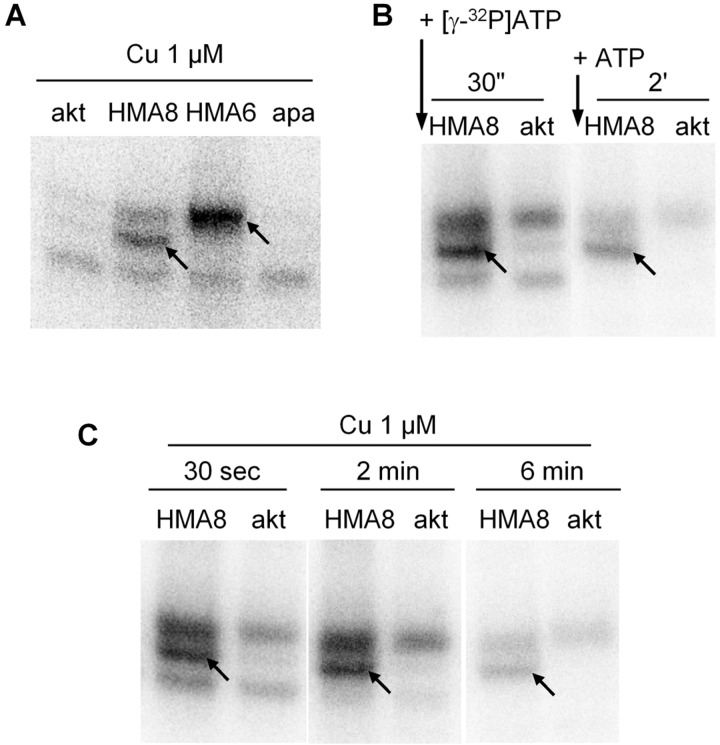
HMA8 is active in Lactococcus membranes (**A**) Phosphorylation from [γ-^32^P]ATP. Membrane preparations containing HMA6 (50 μg) or HMA8/HMA8-AKT/HMA8-APA (100 μg) were phosphorylated under reducing conditions (500 μM Na_2_SO_3_) in the presence of 1 μM CuSO_4_ at pH 6. Phosphorylation from ATP was performed as described in ‘Experimental’ section. (**B**) Isotopic dilution experiments. Membranes containing HMA8 and HMA8-AKT (150 μg) were incubated with 1 μM CuSO_4_ and 1 μM [γ-^32^P]ATP at pH 6. After 30 s, an aliquot was taken and acid-quenched. On the remaining sample, ATP concentration was increased to 10 μM by addition of cold ATP and the reaction was stopped after 2 min. (**C**) Kinetics experiments. Phosphorylation from ATP was performed in the presence of 1 μM CuSO_4_ at pH 6 on membranes containing HMA8 and HMA8-AKT (150 μg). The reaction was stopped at different times (30 s to 6 min) after [γ-^32^P]ATP addition. The arrows indicate HMA6 and HMA8 phosphorylation signals. Akt and apa correspond to the membranes containing the mutated proteins HMA8-AKT and HMA8-APA.

To check that HMA8 was cycling, we performed two types of experiments. One consisted in diluting [γ-^32^P]-ATP with a 10-fold concentrated cold ATP within the reaction. If HMA8 cycles, lowering the specific radioactivity of ATP should decrease the intensity of HMA8 phosphorylation signal, what is shown in Figure 3(B). This decrease cannot be solely attributed to the dephosphorylation of intermediates formed at the beginning of the reaction where specific radioactivity of ATP was high, since as shown in Figure 3(C), over 2-min reaction in these conditions, the phosphorylation signal is nearly constant.

Another way to assess the functionality of HMA8 was to let the reaction run for several minutes so as to reduce ATP concentration. If HMA8 cycles, the ATP concentration should decrease over the time leading to a lower HMA8 phosphorylation level, what is shown in Figure 3(C). Together, these two experiments show that HMA8 is active in Lactococcus membranes and forms a transient phosphorylated intermediate in the presence of ATP and Cu^+^.

### HMA6 and HMA8 display different apparent affinities to Cu^+^

As previously published [29] and shown in the present study (Figure 4A, right panel), HMA6 phosphorylation from ATP is only observed under reducing conditions (obtained by the use of 500 μM Na_2_SO_3_), upon addition of Cu (compare lanes 7 and 8). In these conditions, HMA6 phosphorylation is totally inhibited by a Cu chelators mix containing 1 mM BCA plus 0.1 mM BCS (lane 9). In similar experimental conditions, the behaviour of HMA8 (Figure 4A, left panel) obviously differs from that of HMA6. Indeed, HMA8 phosphorylation is observed in the absence of any added metal (lane 1). It is sensitive to the BCA/BCS mix (lane 2) but not to EGTA (lane 3) a broad range divalent ions chelator, suggesting that the contaminating metal present in the assay and able to activate the transporter is Cu^+^. We tested this hypothesis in performing ICP-MS measurements and found that Cu concentrations, proportional to the amount of membrane used, reached 1.09±0.27 μM in HMA8 assays (approximately one-third of that value in HMA6 assays). To know whether the phosphorylation of HMA8 was maximal at this contaminating Cu concentration, we carried out experiments at different added concentrations of Cu^+^, ranging from 0.5 to 50 μM. These experiments showed that HMA8 phosphorylation was almost achieved at contaminating Cu^+^ (Figure 4B), suggesting an apparent affinity of HMA8 for Cu^+^ in the sub-micromolar range. Note that when experiments were performed in the presence of other metal ions, neither Zn^2+^ (Supplementary Figure S2, lanes 7–9) nor Cd^2+^, Co^2+^ and Mn^2+^ (result not shown) altered the phosphorylation level of HMA8, in agreement with the absence of effect of EGTA.

**Figure 4:**
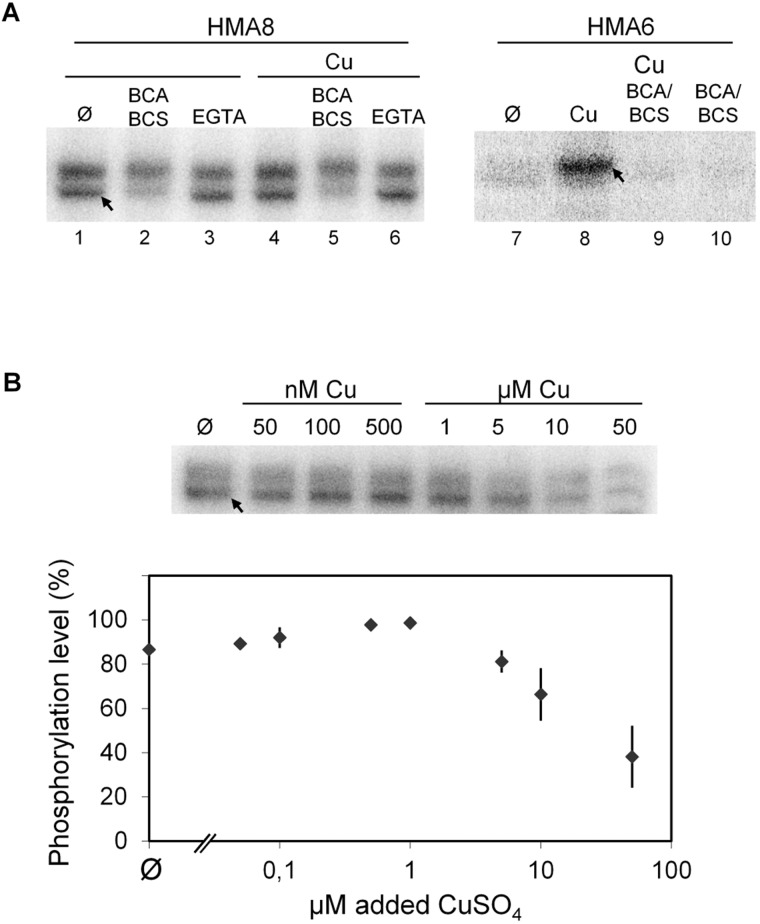
Cu dependence of HMA8 phosphorylation from [γ-^32^P]ATP (**A**) Membrane preparations containing HMA8 (150 μg) or HMA6 (50 μg) were phosphorylated under reducing conditions (500 μM Na_2_SO_3_) without added metal (Ø, lanes 1–3, 7), with CuSO_4_ (1 μM lanes 4–6 and 5 μM lanes 8, 9). In each condition, BCA/BCS (lanes 2, 5, 9 and 10) or EGTA (lanes 3, 6) was added as indicated in the figure. (**B**) Lactococcus membranes containing HMA8 were phosphorylated under reducing conditions with various concentrations of CuSO_4_. Quantifications (lower panel) were made using the Optiquant software (PerkinElmer Life Sciences). Hundred percent corresponds to the maximum phosphorylation level of HMA8. Values correspond to the mean of four independent experiments. Ø corresponds to the values obtained without addition of Cu in the assay. Error bars indicate S.D.

The comparison of the apparent affinities of HMA6 and HMA8 for Cu^+^ is made difficult by the absence of phosphorylation data for HMA8 at Cu^+^ concentrations below 1 μM. However, taking into account the contaminating concentration of Cu measured in HMA6 phosphorylation assays, HMA8 phosphorylation is maximal at a lower Cu^+^ concentration than that of HMA6, suggesting that HMA8 would have a higher apparent affinity for Cu^+^ than HMA6. In order to more accurately measure HMA8 apparent affinity for Cu^+^, we tried to lower contaminating Cu in our samples by prolonged incubations of Lactococcus membranes with chelators (BCA/BCS/EGTA) followed by extensive washings. However, none of these treatments totally switched off HMA8 phosphorylation (result not shown), preventing us to precisely determine the apparent affinity value of HMA8 for Cu^+^.

The inhibition of the phosphorylation at elevated Cu^+^ and Ag^+^ concentrations was previously observed for HMA6 [29]. Although less pronounced, this inhibitory effect was also observed here for HMA8 (Figure 4B; Supplementary Figure S2B). Divalent metals have no inhibitory effect on HMA8 phosphorylation (Supplementary Figure S2B).

### In the presence of ATP, HMA8 would accumulate in a ‘metal occluded phosphorylated form’

Assuming that the intensity of HMA8 phosphorylation is the one measured in the membranes containing HMA8 subtracting from the background signal detected in the same experimental conditions in membranes containing the AKT mutant, BCA/BCS treatment prior to phosphorylation reduces by nearly 40% the phosphorylation level of HMA8 at pH 6 and 7 (Figure 5A). As shown in Figure 3 by kinetics and isotopic dilution experiments, HMA8 is active in Lactococcus membranes. However, HMA8 phosphorylation is only partially sensitive to concentrations of Cu chelators (Figures 4A and 5A) that almost totally abolish HMA6 phosphorylation (Figure 4A). In order to characterize in more details the phosphorylated intermediates of HMA8 formed in the presence of ATP, we tested the effect of ADP on phosphorylation. As shown for many P-ATPases, ADP induces the dephosphorylation of the ‘E~P.Me’ intermediate (step 2 of the catalytic cycle, Supplementary Figure S1). As shown in Figure 5B (lanes 1 and 2), the phosphorylation of HMA8 is almost constant between 1- and 2-min reaction. Addition of the BCA/BCS mix after 1-min reaction and during 1 min (Figure 5B, lane 3 and Figure 5C), leads to ~35% reduction in HMA8 phosphorylation level. This inhibition rate is in the same order as the one measured when the BCA/BCS mix is added prior to the reaction (Figure 4A). Addition of ADP simultaneously to the BCA/BCS mix reduces by 65% HMA8 phosphorylation (Figure 5B, lane 4 and Figure 5C ‘BCA/BCS-ADP’). The effect of ADP is significant but weaker than the one observed on HMA6 where ADP almost totally erases the phosphorylation signal formed from ATP [29]. One hypothesis to explain these results is that approximately one-third of the phosphorylated HMA8 which dephosphorylates rapidly would correspond to the ‘E-P’ intermediate, approximately one-third sensitive to ADP would correspond to the ‘E~P.M’ intermediate. The phosphorylated form insensitive to both ADP and BCA/BCS mix would correspond to what we called ‘metal occluded phosphorylated intermediate’. In this intermediate, the metal would be blocked into the transport site slowing down the evolution towards dephosphorylation.

**Figure 5:**
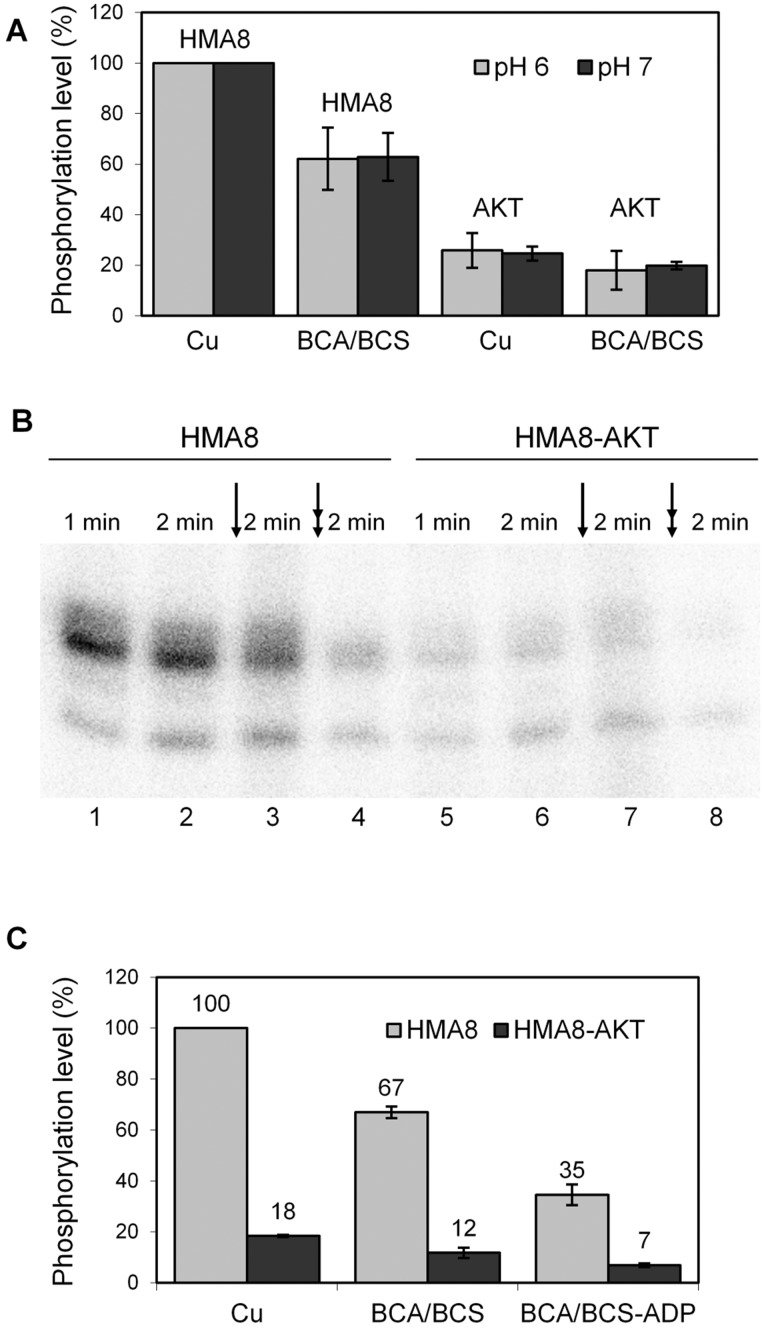
ADP-induced dephosphorylation of HMA8 (**A**) Lactococcus membranes expressing HMA8 (HMA8) or the inactive enzyme HMA8-AKT (AKT) were phosphorylated from ATP in the presence of 1 μM CuSO_4_ (Cu) or 1 mM BCA/100 μM BCS (BCA/BCS) at pH 6 or pH 7. Quantifications were made using the Optiquant software (PerkinElmer Life Sciences). Hundred percent corresponds to the maximum phosphorylation level of HMA8. Values correspond to the mean of five independent experiments. Error bars indicate S.D. (**B**) Lactococcus membranes containing HMA8 (150 μg) and HMA8-AKT (150 μg) were phosphorylated from ATP at pH 6 in the presence of 1 μM CuSO_4_. Following 1 min reaction, the sample was acid-quenched (lanes 1 and 5) or incubated 1 min more either without any addition (lanes 2 and 5) or with BCA/BCS (single arrow, lanes 3 and 7) or with BCA/BCS plus 1 mM ADP (double arrow, lanes 4 and 8) prior to acid-quenching. Samples were submitted to an acidic SDS/PAGE and phosphorylation was detected using a phosphoimager. Quantifications (**C**) were made using the Optiquant software. 100% corresponds to the phosphorylation level of HMA8 in presence of Cu following 2 min reaction (lane 2). Values correspond to the mean of three independent experiments. Error bars indicate S.D.

### HMA8 and HMA6 display different dephosphorylation kinetics

The results presented so far suggest that HMA8 could have enzymatic properties distinct from those previously revealed on HMA6 [29]. To more deeply understand the difference between the two transporters, we compared their dephosphorylation kinetics in the presence of the BCA/BCS mix and at 4°C to slow down the reaction. As illustrated in Figure 6(A) and quantified in Figure 6(B), the dephosphorylation process is biphasic for the two transporters but whereas it is complete for HMA6, almost reaching the background level at 10 min (compare lanes 5 and 7), it significantly remains partial in the case of HMA8, even after addition of 1 mM ADP (Figure 6B, double arrow). This somehow corroborates the results shown in Figures 4 and 5.

**Figure 6:**
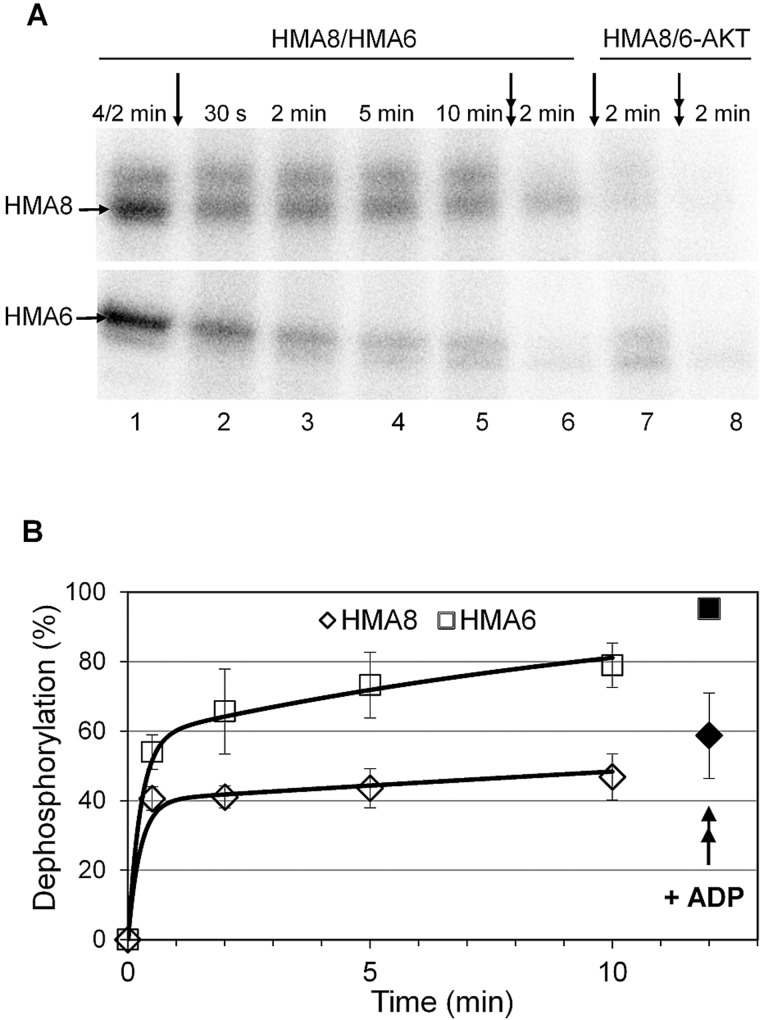
Dephosphorylation kinetics in presence of Cu chelators This experiment was performed at 4°C, at pH 6. Lactococcus membranes containing HMA8, HMA6, HMA8-AKT and HMA6-AKT were incubated under reducing conditions (500 μM Na_2_SO_3_) with Cu and 1 μM [γ-^32^P]ATP. After 2 min (for HMA6) or 4 min (for HMA8) an aliquot was taken and acid-quenched (lane 1). On the remaining sample, a mix of BCA/BCS was added and the reaction was stopped at the indicated times (single arrow, lanes 2–5 and 7). After 10 min of incubation with BCA/BCS, 1 mM ADP was added to the reaction for 2 min more (double arrow, lanes 6 and 8). All the experiments described in this figure were performed with 1 μM CuSO_4_ for the phosphorylation of Lactococcus membranes containing HMA8/HMA8-AKT (150 μg) or with 5 μM CuSO_4_ for the phosphorylation of Lactococcus membranes containing HMA6/HMA6-AKT (50 μg). Quantifications (**B**) were made using the Optiquant software. Hundred percent dephosphorylation correspond to the intensity of the background level as measured on the HMA8-AKT mutant. Values correspond to the mean of three independent experiments. Error bars indicate S.D.

One can hypothesize that the first phase, similar for HMA6 and HMA8, corresponds to the fast ‘E-P’ to ‘E’ dephosphorylation (Supplementary Figure S1, step 4). The second phase, obviously different between the two transporters could represent the dephosphorylation of two forms: the ‘E~P.M’ (ADP sensitive) and a ‘E-P.[M_occluded_]’ (ADP insensitive) forms. The significant amount of remaining phosphorylation observed for HMA8, even after ADP addition, shows that this transporter accumulates this later form and that metal release is a particularly limiting step in HMA8. This does not seem to be the case for HMA6 where dephosphorylation, although biphasic is almost complete after 10 min. A similar kinetics (a biphasic dephosphorylation process in the presence of BCS with 50% of residual phosphorylation) has already been observed for the human ATP7A Cu-ATPase [43].

### Modelling of HMA6 and HMA8 and docking of plastocyanin

Hypothesizing that metal deocclusion was particularly slower in HMA8 than in HMA6, we searched molecular explanations in the structure of the two transporters. For that, HMA6 and HMA8 were modelled based on the structure of the *L. pneumophila* Cu^+^-ATPase LpCopA [11,44] with the MODELLER program [33] and their electrostatic potential maps were calculated (see ‘Methods’). A very clear difference appears between HMA6 and HMA8 Cu-releasing cavities, the former predominantly exhibiting a positive charge surface whereas the latter displays a rather negative one (Figure 7A). Such difference might explain the observed dephosphorylation kinetics (see ‘Discussion’).

**Figure 7:**
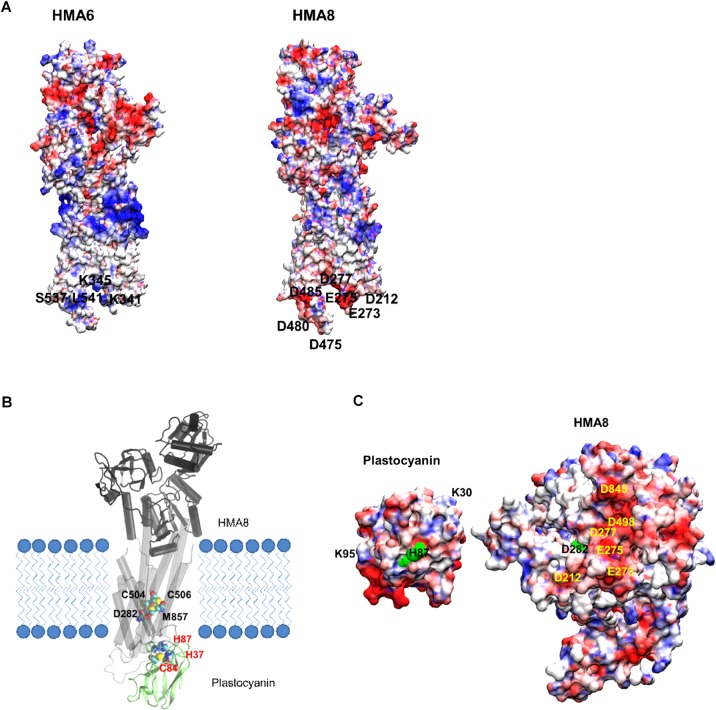
Modeling of HMA6-HMA8 and docking of plastocyanin (**A**) Electrostatic potential maps of HMA6 and HMA8 were modelled from the structure of LgCopA (PDB code 4BBJ) [11] with the MODELLER program [33]. The HMs are oriented with kinked helix III in front. The figure highlights charge differences between surface residues of HMA6 and HMA8 in the inner cell region. HMA6 presents positively charged residues (coloured blue) such as Lys^341^ and Lys^345^ which align (both in sequence and in structure) with the negatively charged residues (coloured red) Glu^273^ and Asp^277^ of HMA8. Notice that the patch of residues Ser^537^ to Leu^541^ in HMA6 exposes their backbone amine group (positive polarity) in a region of the sequence which structurally aligns with the negatively charged Asp^480^–Asp^85^ region in HMA8. (**B**) Docking of PC [modelled from the structure of poplar PC (PDB code 4DP7) [34]] to HMA8 using the program HADDOCK [38]. Residues Cys^84^ and His^87^ in PETE2 and Asp^282^, Met^857^ in HMA8 were defined as active residues for HADDOCK meaning that the program favours the presence of these residues at the docking interface. The proteins are shown in cartoon representation inside a phospholipid membrane. The Cu-binding site in PC constituted of amino acids His^37^, Cys^84^ and His^87^ and a possible Cu route inside HMA8 leading from Cys^504^, Cys^506^ (CPC motif) to Met^857^ and Asp^282^ are highlighted in CPK. (**C**) Electrostatic potential maps for PC and HMA8. Proteins were docked as shown in (**B**) then rotated so as to show their interface residues. Positively charged patches (coloured blue) appear in PC due to the presence of Lys^30^ or Lys^95^, for instance, whereas interfacial residues in HMA8 are mostly negatively charged (red) with lots of aspartic and glutamic acid residues (Asp^212^, Glu^273^, Glu^275^, Asp^277^, Asp^498^, Asp^845^…). Asp^282^ in HMA8 and His^87^ in PC proposed to bind Cu along its way outside the ATPase are shown in green CPK representation. Figures and molecular surfaces were drawn with program VMD [37] and electrostatic maps calculated with APBS [36] within VMD.

To gain further information on the possible interaction between HMA8 and PC, we performed docking calculations in the same way as done in a previous structural study between the metallochaperone HAH1 (human ATX1 homologue) and the human Menkes ATPase [45]. *Arabidopsis* PC isoform modelled from the 1.08 Å crystal structure of the popular PC was docked to the HMA8 model as described above. Docking calculations and electrostatic potential maps showed that PC could interact with HMA8 with its His^87^ facing the Cu-release site of the transporter (Figures 7B and 7C).

## DISCUSSION

The localization of HMA8 in the thylakoids was evidenced first in *Arabidopsis* by transient expression of a truncated form fused to GFP [21] and later in soybean chloroplasts by western blot experiments [46]. As a scarce protein, HMA8 had never been detected by proteomic analyses targeted to thylakoids until our recent work, using sub-thylakoid fractionation, which led to HMA8 detection by MS in the stroma-lamellae [47]. HMA6 and HMA8 are considered as the main way to provide Cu to the PC even though reverse genetic approaches have suggested the existence of other Cu import pathways both in the chloroplast envelope and in the thylakoids. Since Cu is essential for PC, its targeting to the thylakoid lumen has to be finely tuned. To get more insights into the regulation of Cu homoeostasis, we compared the enzymatic properties of HMA6 and HMA8 when inserted in a biological membrane. Since HMA6 was already biochemically characterized in Lactococcus and yeast [29], we performed similar analyses on HMA8.

We show in the present paper that HMA8 expressed in Lactococcus membranes behaves as a classic P-ATPase, forming a transient phosphorylated intermediate in the presence of ATP and the transported ion, Cu^+^. In its membrane fraction, HMA8 is almost fully phosphorylated from ATP in the presence of contaminant Cu^+^ whose concentration, as measured by ICP-MS, is ~1 μM. Nearly the same metal concentration was detected in membrane fractions containing HMA8-AKT (the non-functional form of HMA8) or HMA6 (10 times more abundant than HMA8), showing that the amount of contaminant Cu^+^ is related neither to the functionality of the transporter nor to its abundance. The concentration of contaminant Cu^+^ available for HMA6 and HMA8 is probably lower than that measured by ICP-MS since part of the metal is probably membrane- or protein-bound. In our experimental conditions, almost all HMA8 molecules are saturated with contaminant Cu^+^ as deduced from the minor effect of CuSO_4_ addition on the activation of the phosphorylation (Figure 4B). This is not the case for HMA6 which displays almost no phosphorylation from ATP in the presence of contaminant Cu^+^ (Figure 4B), suggesting that HMA6 has a weaker affinity for Cu^+^ than HMA8. In the course of our work, it was shown that purified HMA6 and HMA8, in detergent and without their N-terminal MBD, have similar affinity for Cu^+^, in the sub-micromolar range [26]. Since deletion of the N-terminal MBD domain can have an impact on the affinity of Cu^+^-ATPases for Cu^+^ [43,48], it is thus difficult to compare results obtained in this study to ours.

Like for HMA6, HMA8 phosphorylation from ATP is inhibited by high Cu concentrations. It is well documented on P-ATPases that high concentrations of the transported ion can prevent ion release from the low-affinity transport site and consequently inhibit the ATPase and the transport activities. According to the commonly admitted P-ATPase catalytic cycle, blocking ion release will stabilize the ion-bound phosphorylated intermediate. This can be illustrated with the Ca^2+^-ATPase SERCA1a (sarcoplasmic reticulum calcium ATPase) [49], the Na^+^,K^+^-ATPase [50] and also the P_IB_-ATPases like CadA [39] and the *Arabidopsis* Zn^2+^-ATPase HMA4 [51] whose phosphorylation levels are stable over a wide concentration range above the *K*_d_ value of the transported ion. In this context, the inhibition by high Cu concentrations of the phosphorylation from ATP of some eukaryotic Cu^+^-ATPases (ATP7A [43,52]; HMA6 [29]; Ccc2p, Lowe, J., Cuillel, M. and Mintz, E., unpublished data; HMA8, present work) is rather unexpected and the reasons of this inhibition are still unknown.

Cu seems tightly bound to HMA8. Indeed, the strong Cu chelators, BCA and BCS, added either prior to the phosphorylation assay (Figures 4A and 5A) or within membranes preparation (result not shown) only partially reduce the phosphorylation level of HMA8 (higher concentrations of chelators were found to have deleterious effects on the sample and could not be tested). On the contrary, the same chelators mix completely abolishes HMA6 phosphorylation from ATP in the presence of 5 μM added Cu (Figure 4A). A significant basal activity in the absence of added Cu and a persistent activity even in the presence of Cu chelators have already been reported for other Cu^+^-ATPases like Ccc2p [12], CopA [9] and ATP7B [53]. The basal activity in absence of added Cu was attributed to contaminant Cu (as proposed in the present study for HMA8) but the explanation for the residual activity in presence of Cu chelators is less clear especially because BCS and BCA have a very high affinity for Cu^+^ (logβ_2_ ~20 and 17 for BCS and BCA respectively; [54]).

The differences between HMA6 and HMA8 in terms of dephosphorylation kinetics as depicted in Figure 6 led us to look for structural differences between the catalytic domains of the two transporters and especially as significant differences exist also in the primary sequence of their A, N and P domains. Hence, the three domains of HMA6 and HMA8 were expressed in *E. coli*, purified and then submitted to crystallization assays. So far, only the 3D-structure of the N domain of HMA8 has been obtained at 1.75Å resolution preventing any structural comparison between the catalytic domains of HMA6 and HMA8 (result not shown).

By analogy with the two human Cu^+^-ATPases ATP7A and ATP7B, the functional differences between HMA6 and HMA8 might be related to distinct cellular roles. Indeed, it was shown that ATP7B, which displays a high apparent affinity for Cu and a slow catalytic activity, has a key role in delivering Cu to the Golgi resident Cu-dependent ferroxidase whereas ATP7A, which displays a low apparent affinity for Cu and high turnover rate, is essential for the maintenance of the adequate intracellular Cu concentration [55]. To some extent, HMA6, which displays a lower apparent affinity for Cu and a higher turnover rate than HMA8, might be the plant counterpart of ATP7A, regulating Cu concentration in the stroma, whereas HMA8 would have more precise role in delivering Cu to PC. This difference between HMA6 and HMA8 is not found in the study of Blaby-Haas et al. [26]. However, in their case, they worked on purified proteins, truncated of their N-terminal MBD domain and measured their ATPase activity in the presence of detergent. In our case, we focused on phosphorylated intermediates of full-length proteins, in a membrane environment. These two different approaches on different forms of the same transporters make difficult to compare the results obtained from both studies.

The difference between HMA6 and HMA8 in terms of dephosphorylation kinetics raises the question of how the characteristics of the Cu-receiving compartments (stroma for HMA6; thylakoid lumen for HMA8) and the nature of the Cu^+^ acceptors (formally unknown for HMA6; PC for HMA8) impact on the activity of the transporters. Under illumination, an important difference between the two Cu releasing compartments is the pH, ~7.8 in the stroma but much more acidic (~pH 5.5) in the thylakoid lumen. To understand how pH might affect the protonation of residues involved in Cu release, we modelled the structures of HMA6 and HMA8 (Figure 7A) and evidenced differences in the electrostatic potential of their Cu-releasing cavities. Indeed, HMA6 exhibits a positive charge surface in its Cu-releasing cavity whereas HMA8 displays a rather negative one. Then, in our *in vitro* assays, it could be that, due to electrostatic interactions and/or to the absence of a Cu^+^ acceptor, Cu release from HMA8 is slowed down, leading to the accumulation of a particular intermediate state we called E-P.[M_occluded_] form and explaining the low dephosphorylation kinetics of the transporter. On the other hand, Cu^+^ release from HMA6 would be favoured by charge repulsion.

In *Arabidopsis*, PC is synthesized in the cytosol as a precursor and then translocated into the thylakoid lumen where it is processed to its mature form and acquires Cu [56]. Due to its function in the photosynthetic electron transfer chain, PC cycles between oxidized (PC-Cu^2+^) and reduced (PC-Cu^+^) forms, catalysing electron transfer from the cytochrome *f* in the *b*_6_/*f* complex to P700^+^ in photosystem I. So far, the form of Cu (1+ compared with 2+) that ends PC maturation in the thylakoid lumen is not known but in native and unfolded forms, PC affinity for Cu^+^ is 3 orders of magnitude higher than for Cu^2+^ [57,58]. As HMA8 transports Cu^+^, we hypothesized that, after cleavage by a lumen thylakoid peptidase, apo-PC acquired Cu^+^ directly from HMA8 to form the holoprotein. In that case, the neosynthesized PC-Cu^+^ would be ready to transfer an electron to PSI. Our docking and electrostatic potential maps calculations seem to corroborate this hypothesis. Indeed, PC and HMA8 interaction could be favoured by electrostatic forces allowing the Cu release cavity of HMA8 to face His^87^ of PC (Figures 7B and 7C).

Cu delivery to the thylakoid lumen is a priority for plant cells. When Cu availability is reduced, plants adapt at the transcriptional level through the Cu-responsive transcription factor SPL7 (squamosa Promoter binding protein Like 7) by down-regulating non-essential Cu-proteins like Cu/Zn SOD for example [59] and at the post-translational level by stabilizing HMA8, a mechanism related to PC level (more precisely to the PETE2 isoform) and the chloroplast caseinolytic protease system [60,61]. These processes ensure the optimal use of Cu and its delivery to PC via HMA6 and HMA8. The biochemical properties of HMA6 [29] and HMA8 (present work) are thus in good agreement with this model. Besides these two regulatory mechanisms identified to facilitate the flow of Cu to the thylakoid lumen under limiting Cu, the enzymatic properties of HMA8 (high affinity for Cu- and PC-dependent turnover rate) may be an additional way to regulate the flux of Cu into PC and thus to regulate the photosynthetic electron flow.

## Abbreviations

BCA: bicinchoninic acid
BCS: bathocuproine disulfonate
ER: endoplasmic reticulum
HRP: horseradish peroxidase
ICP-MS: inductively coupled plasma mass spectrometer
MBD: metal-binding domain
PC: plastocyanin
SOD: superoxide dismutase
TM: transmembrane segment
